# Very low-calorie diet in candidates for bariatric surgery: change in body composition during rapid weight loss

**DOI:** 10.6061/clinics/2019/e560

**Published:** 2019-03-05

**Authors:** Marcela Pires Serafim, Marco Aurelio Santo, Alexandre Vieira Gadducci, Veruska Magalhães Scabim, Ivan Cecconello, Roberto de Cleva

**Affiliations:** I Unidade de Cirurgia Bariatrica e Metabolica, Disciplina de Cirurgia Gastrointestinal, Faculdade de Medicina FMUSP, Universidade de Sao Paulo, Sao Paulo, SP, BR

**Keywords:** Body Composition, Severe Obesity, Bariatric Surgery, Weight Loss, Diet

## Abstract

**OBJECTIVE:**

To analyze the changes in the body composition of morbidly obese patients induced by a very low-calorie diet.

**METHODS:**

We evaluated 120 patients selected from a university hospital. Body composition was assessed before and after the diet provided during hospitalization, and changes in weight, body mass index, and neck, waist and hip circumferences were analyzed. Bioimpedance was used to obtain body fat and fat-free mass values. The data were categorized by gender, age, body mass index and diabetes diagnosis.

**RESULTS:**

The patients consumed the diet for 8 days. They presented a 5% weight loss (without significant difference among groups), which represented an 85% reduction in body fat. All changes in body circumference were statistically significant. There was greater weight loss and a greater reduction of body fat in men, but the elderly showed a significantly higher percentage of weight loss and greater reductions in body fat and fat-free mass. Greater reductions in body fat and fat-free mass were also observed in superobese patients. The changes in the diabetic participants did not differ significantly from those of the non-diabetic participants.

**CONCLUSIONS:**

The use of a VLCD before bariatric surgery led to a loss of weight at the expense of body fat over a short period, with no significant differences in the alteration of body composition according to gender, age, body mass index and diabetes status.

## INTRODUCTION

Morbid obesity is a risk factor for higher mortality during the preoperative period of bariatric surgery 1, and it requires care that includes pharmacological monitoring, anesthesia 2 and standard ventilator use 3. Several benefits have been demonstrated for weight loss between 5 and 10% 4 through conventional clinical treatments, such as a significant decrease in visceral adipose tissue 2,4 and liver volume 2 and decreases in risk factors 3, surgical risk 5,6, blood loss 2, length of surgery 4,6 and length of hospital stay 7. Given this evidence, weight loss prior to bariatric surgery is recommended 7.

In dietary intervention, calorie restriction is the most important factor 8. The very low-calorie diet (VLCD) contains between 400 to 800 kcal/day and is indicated for individuals with a BMI >40 kg/m^2^ who need to control their comorbidities 9,10. Its impact on the change in body composition in morbidly obese 11 and its benefits during the perioperative period of bariatric surgery have been shown in several studies 3,6,12,13. At our institution, patients with BMI>50 kg/m^2^ had a weight loss approximately of 10% in 7.7 weeks with a VLCD 14. Although body weight represents an important factor in the nutritional evaluation of these individuals, only this parameter can omit the physiological processes that comprise the weight loss, such as the alteration of body compartments. Thus, the measurement of body compartments and their changes presents a great challenge; nonetheless, it is extremely important in nutritional assessment to achieve successful surgery and minimize nutritional complications 15,16. In addition, obesity is characterized by large changes in body composition compared to eutrophy; in addition to having a higher volume of adipose tissue, obese individuals present a general increase in body water, with a higher volume of extracellular water (ECW) in relation to intracellular water (ICW) 17, and increase in total body water (TBW) 18. These factors make it difficult to accurately diagnose each compartment and its alterations since even body composition assessment methods that are considered gold standards, such as double X-ray absorptiometry (DEXA), air displacement plethysmography (ADP), magnetic resonance and computed tomography (CT), are limited by the physical capacity of the equipment 16; additionally, their accuracy is reduced by high volumes body water, and they are expensive and inconvenient to use in clinical practice 19. Among the most widely used methods for assessing body composition, bioimpedance (BIA) is considered safe, fast, simple, applicable to clinical practice and research and highly reproducible 20. When used alone, BIA seems to be more strongly associated with the body fat (BF) percentage than the BMI 19, but, for severe obesity, equations are inadequate for estimating body composition over time; therefore, caution should be used when interpreting these estimates and other methods for assessing body composition 21. However, a clinical study conducted in Brazil validated an equation for estimating % BF through BIA based on the amount of BF obtained by ADP; this equation increased the precision of BF estimation compared to the equation used by the device itself and reduced the limitations of BIA use by extending it to the population with BMI >34 kg/m^2^ 22. Considering that a VLCD is an option for optimizing weight loss before surgery, information about its short-term impact on patients' body composition will allow the application of new procedures and early intervention, thus minimizing possible nutritional complications. Consequently, the objective of this study was to analyze the alterations of the body composition of bariatric surgery candidates after weight loss with VLCD.

## METHODS

An observational prospective study evaluating body composition was conducted in patients undergoing bariatric surgery between June 2013 to September 2014 at the Bariatric and Metabolic Surgical Unit of a hospital medical school. Patients with a pacemaker, acute or chronic diseases associated with excessive water retention (congestive heart failure, chronic renal failure, or liver failure), subjects with weight loss greater than 5% before hospitalization and those who did not agree to participate in this study were excluded.

The present study was approved by the ethics committee (protocol n° 00984812.5.0000.0068). The study did not interfere with the provision of care and did not include any type of intervention. Informed consent was obtained from each patient before their participation in the study.

Patients older than 18 years who were hospitalized for a minimum of 7 days and a maximum of 10 days before surgery were included in this study. This hospitalization period is a routine practice in our service for patients who require considerable weight loss or highly complex tests that cannot be conducted on an outpatient basis.

### Study protocol

Nutritional assessment of body composition was performed at two timepoints: at admission and before surgery (after 7-10 days on the VLCD). The results were classified according to gender, age (<59 years and ≥60 years), BMI [≥40 (morbid obesity) and >50 kg/m^2^ (super obesity)] 23 and the presence of diabetes mellitus (DM).

### Anthropometric evaluation

An electronic scale was used to measure weight (model W 300A, Welmy^®^, Brazil), and a stadiometer coupled to the scale was used to measure height. The nutritional evaluation was performed in the morning, while the patient was in bed and in a fasting condition. Neck circumference (NC), waist circumference (WC) and hip circumference (HC) were measured using an inelastic tape measure 2 meters in length.

### Body composition

For the analysis of body composition, a bioimpedance analyzer Model 310 was used. To estimate BF, the following equation was used: BF (kg) = 23.25+ (0.09 x resistance in ohms) + (1.00 x weight in kg) - (0.08 x height in meters) + (0.13 x age in years). To obtain the fat-free mass (FFM) value, body weight (kg) - BF (kg) was calculated and compared with the estimation method. For the TBW analysis, the result obtained with the equipment's equation was used; thus, this compartment was analyzed in isolation.

### Diet

Consistent with the established routine for nutritional assistance during the preoperative hospitalization period, a 600 kcal diet containing on average 20% protein, 20% fat and 60% carbohydrates was offered.

### Statistical methods

For the data analysis, R 3.0.2 was used (core team). We considered *p*<0.05 statistically significant. The results are reported as the mean, standard deviation, and minimum and maximum values. To compare the body composition data before and after the VLCD, Student's t test and the Mann-Whitney test were used. To analyze the changes in body composition according to gender, age, BMI and the presence of DM, multiple regression analysis was performed. Logistic regression was performed to identify the prevalence of each group.

## RESULTS

**Figure 1:**
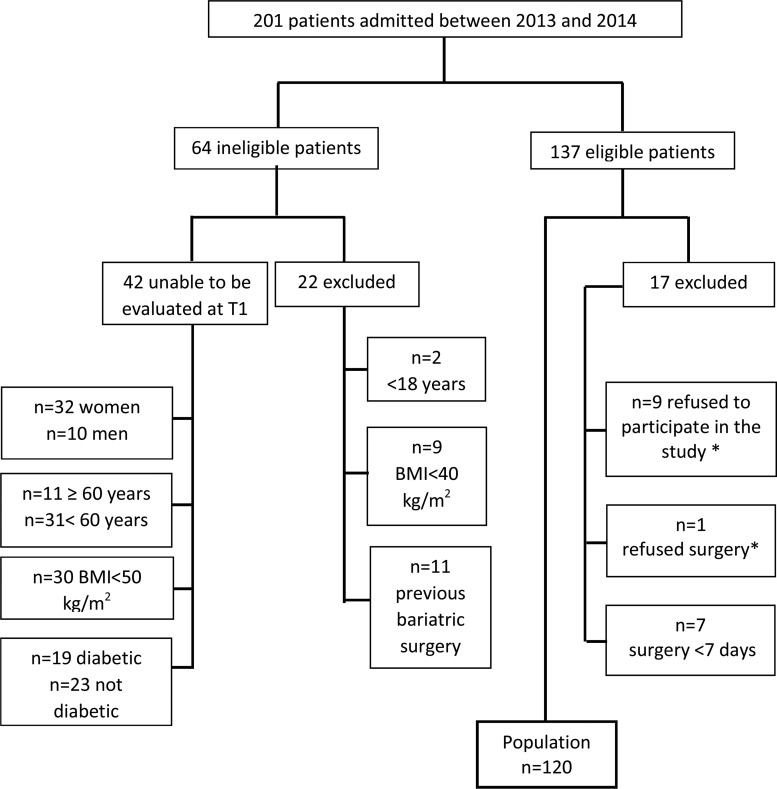
Candidates for bariatric surgery admitted to the hospital medical school between July 2013 and September 2014 and the composition of the study population. * Motive not questioned; ** Diagnosis / reason for admission: n=5 incisional hernia, n=2 malnutrition, n=3 ring removal, n=1 intestinal stenosis.

The sample comprised 120 patients; 76% (n=91) were female, 8% (n=9) were ≥60 years, 45% (n=54) had a BMI ≥50 kg/m^2^, and 30% (n=35) had a diagnosis of DM. The mean preoperative hospital stay was 8.5±1.05 days. Figure 1 describes the characteristics of the patients hospitalized during the study period.

### Analysis of body composition at hospital admission

The characteristics of the bariatric surgery candidates at hospital admission are shown in Table 1. Male patients had larger body circumferences and greater BF, FFM and TBW (*p*<0.05). Additionally, individuals with BMI ≥50 kg/m^2^ presented higher values for body circumference and all body compartments (*p*<0.05). Patients with DM had lower body weight and BMI values (*p*=0.01 and *p*=0.02, respectively), and lower HC and BF values (*p*=0.01 for both).

### Alterations in body composition after the VLCD

**Figure 2:**
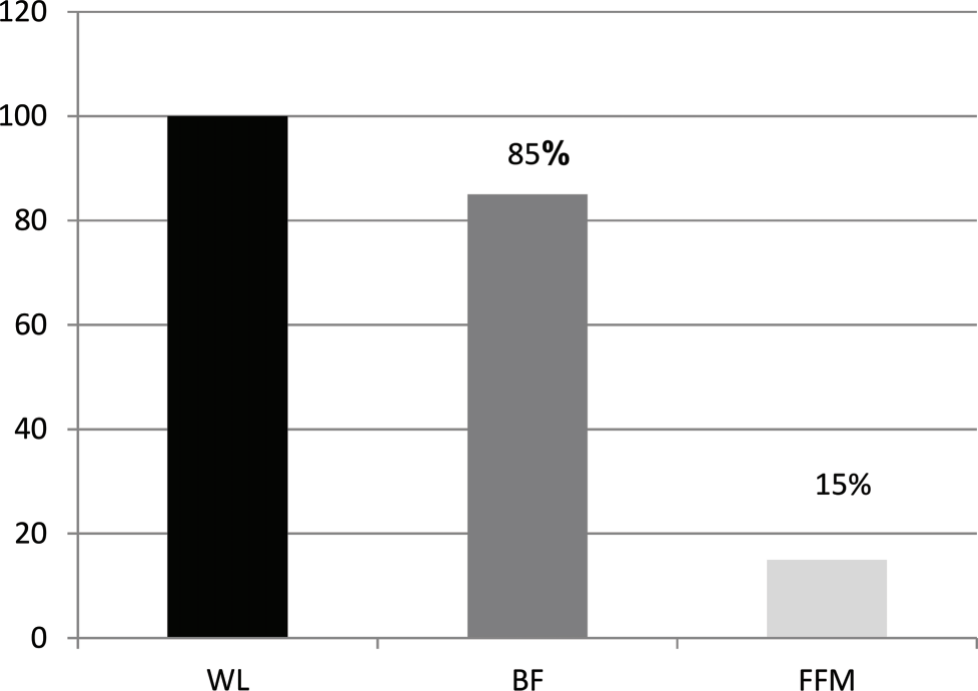
Changes in BF and FFM in relation to weight loss (%) Note: BF, body fat; FFM, fat-free mass; WW, weight Loss.

The bariatric surgery candidates presented a weight loss of approximately 5% (85% due to reduced BF (Figure 2).

The weight loss and BMI reduction were significant (*p*=0.02 and *p*=0.001, respectively). All of the evaluated body circumferences decreased (*p*<0.05 for all measures), as did BF and FFM (*p*=0.01 for both) (Table 2).

A higher percentage of weight loss (*p*=0.03) and greater reductions of WC, HC, BF and FFM were found in elderly patients. In diabetics no significant difference was found for the changes, although they presented lower weight loss and lower BF reductions. These results are shown in Table 3.

## DISCUSSION

Given the increasing numbers of candidates for bariatric surgery, it is important to select and prepare patients in a way that increases their success after surgery 5. Evidence has demonstrated a positive association between weight loss before surgery and better postoperative results 2,3,5,24-27. In terms of weight loss, diet is an essential factor in routine care for the morbidly obese, and the impact of the VLCD on changes in the body composition of morbidly obese individuals and its benefits during the perioperative period of bariatric surgery have been demonstrated 24,28.

In this study, we observed moderate weight loss in individuals with morbid obesity during a short period of hospitalization. Several authors have also shown the effects of VLCD on weight loss: Faria et al. 28 reported a weight loss of -2.2±2 kg after 7 days on the diet, Merino et al. 29 reported a loss of 6.1±3.3 kg on a diet of 800 kcal for 3 weeks, and Malandrucco et al. 12 reported that a diet of 400 kcal for 7 days led to a weight reduction of 3.6 kg. The results varied according to the type of caloric restriction and duration for which the diet was consumed.

When evaluating body composition with BIA, it is important to consider that the results obtained from the equipment's equation can be influenced by body geometry and water distribution; in obese individuals, BIA may underestimate BF and overestimate FFM 22,30.

Despite this, BIA was the method of choice for this study because it is a more sensitive method for estimating BF in obese patients than weight, height and BMI 21. Furthermore, it has been validated as a measure of body adiposity when compared with reference methods such as DEXA, is more strongly associated with % BF than BMI, and can be used as a tool to measure BF 17. In addition, it is a quick examination that does not depend on prior scheduling (and thus allowed evaluations to be performed within the period that was predefined in the methodology of this study), and it required less mobility and thus can accommodate patients who have locomotor limitations and cannot be taken to another area; these characteristics were indispensable for this study, which was carried out during the inpatient unit routine. This study did not use other methods to evaluate body composition, such as DEXA, ADP or CT, because they are limited by the maximum capacity of the equipment; additionally, these methods may overestimate FFM in this population 16,20, which intensifies the need to use a viable method to evaluate the body composition alterations of these individuals in clinical practice 20.

There are numerous equations available for calculating body composition through the resistance produced by the BIA; however, it is common knowledge that the information included in the device software is not known, especially in relation to its validity for the population study, which leads to errors in the estimation of the body compartments 31. Most of the specific equations that were used to validate BIA for obese patients are unsuitable for individuals with a BMI>34 kg/m^2^ 21,22; consequently, the selection of an adequate equation to evaluate BF in these individuals is crucial to achieving more accurate results 16. For this reason, a equation that has been validated for this population 22 was used to minimize errors in the estimation of body composition with the device.

Regarding body circumference, several studies have shown alterations with the use of VLCD. In terms of the change in NC after the use of VLCD for 10 days, but a -5.9 cm reduction was observed after 12 weeks of diet 32, representing a smaller change compared to the results of the present study. Regarding WC, Leonetti et al. 32 observed a reduction of -8.5 cm after 30 days on the diet, Faria et al. 19 showed reduction of -3.1±7 cm after 14 days on the diet, Merino et al. 29 observed a reduction of -5.5±4.2 cm after 3 weeks, and Malandrucco et al. 12 found that a diet of 400 kcal for 7 days yielded a reduction of -3.4±1.36 cm in WC. The present study observed a smaller reduction in WC compared to these previous studies. Regarding HC, Verhoef et al. 33 and Perez et al. 34, demonstrated reductions of -6.1 cm and -4.5 cm after 8 weeks of a VLCD, respectively, which is a smaller reduction compared to the present study. Changes in body circumference measures did not reduce the risk of cardiometabolic diseases, but the reduction of BF is an important aspect of preparation for bariatric surgery.

The proportion of weight loss represented by BF reduction observed in this study has also been demonstrated by other researchers, and most of the results are consistent with the findings of the present study. Camps et al. 35 analyzed 48 individuals with a mean BMI of 31.4±2.8 kg/m^2^ and found that after 8 weeks of VLCD use, 80% of weight loss was BF. With Verhoef et al. 33 the results showed that 82% of weight loss was related to a decrease in BF, and Siervo et al. 36 showed a BW loss of 4.8% after 10 days on the VLCD, 68.9% of which was BF.

When discussing the assessment of body composition in individuals with morbid obesity, it is important to use methods and parameters that complement the diagnosis and to analyze the evolution of nutritional status. In addition, it is important for the professional to be able to interpret what the test shows while considering that factors such as medication use, hydration, excess BF, and the techniques and equations used can lead to errors in estimation and consequently in the nutritional evaluation of the patient.

The loss of FFM, particularly muscle mass, is a continuing concern, and there is a need for further studies of the use of VLCD in morbidly obese patients using more precise techniques to analyze this compartment.

## CONCLUSION

The use of a VLCD before bariatric surgery led to weight loss at the expense of BF in a short time. There was no significant difference in the change in body composition according to gender, age, BMI and diabetes.

Further studies are needed to more specifically evaluate the body composition – mainly hydration - in this population, and to evaluate the impact of VLCD during the postoperative period for these patients.

## AUTHOR CONTRIBUTIONS

Serafim MP and Santo MA were responsible for the study design, data collection, data analysis and manuscript writing. Gadducci AV was responsible for data collection and manuscript writing. Scabim VM was responsible for data collection and data review. Cecconello I was responsible for data review. de Cleva R was responsible for the analysis and review of the results and manuscript writing.

## Figures and Tables

**Table 1:** Characteristics of the bariatric surgery candidates at admission according to gender, age, BMI and diabetes diagnosis.

	Weight (kg)	BMI (kg/m^2^)	NC (cm)	WC (cm)	HC (cm)	BF (kg/%)	FFM (kg/%)	TBW (L/%)
Men (n=29/24%)	155±3.99	50.6±1.12	51.5±0.78	140.5±2.16	150.0±2.78	75.9±3.28/ 50.7±1.19	76.4±1.39/ 49.3±1.19	70.0±1.71/ 45.7±0.57
Women (n=91/76%)	124.9±1.84	49.1±0.58	42.0±0.46	126.5±1.41	142.0±1.50	64±1.39/ 52.8±0.59	57.4±0.92/ 47.2±0.59	43.3±0.63/ 35.2±0.33
*p value*	0.01*	0.80	0.02*	0.01*	0.11	0.01*/0.02*	0.01*/0.02*	0.02*/0.01*
Age ≥60 years (n=9/8%)	129±5.77	49.5±1.74	43±1.55	133±3.55	146.1±5.31	70.5±4.97/ 51.6±2.29	55.3±3.36/ 48.4±2.29	43.1±3.88/ 38.1±2.03
Age<60 years (n=111/92%)	130±2.05	49.3±0.53	44±0.56	129±1.38	144±1.37	67.3±1.42/ 52.3±0.56	62.7±1.04/ 47.7±0.56	45.7±1.21/ 36.8±0.53
*p value*	0.43	0.21	0.39	0.69	0.69	0.93/0.32	0.15/0.63	0.65/0.63
BMI≥50 kg/m^2^(n=54/45%)	145.0±2.43	53.7±0.39	44.9±0.83	137.0±1.81	152.0±1.43	81.3±1.4/ 54.5±0.58	64.2±1.49/ 45.5±0.58	49.5±1.83/ 35.6±0.78
BMI<50 kg/m^2^(n=66/55%)	117.8±1.98	44.8±0.44	42.8±0.63	123.3±1.47	135.3±1.61	57.2±1.24/ 48.7±0.7	57.7±1.3/ 51.3±0.7	43.2±1.26/ 37.3±0.69
*p value*	0.01*	0.01*	0.01*	0.01*	0.01*	0.03*/0.02*	0.03*/0.01*	0.03*/0.08
DM (n=35/29%)	118.2±3.6	47.3±1.0	43.8±0.93	125±2.39	137±2.55	61.9±2.44/ 51.4±1.02	56.8±1.75/ 48.6±1.02	43.2±2.09/ 37.3±0.88
No DM (n=85/71%)	132±2.22	50.2±0.58	44±0.65	131.5±1.54	148±1.49	70.2±1.58/ 52.6±0.64	63.9±1.19/ 47.4±0.64	46.5±1.38/ 36.4±0.63
*p value*	0.01*	0.02*	0.92	0.15	0.01*	0.01*/0.23	0.07/0.23	0.13/0.20

Note: DM: diabetes mellitus; BMI: body mass index; NC: neck circumference, WC: waist circumference; HC: hip circumference; BF: body fat; FFM: fat-free mass; TBW: total body water. *p<0.05.

**Table 2:** Alteration of body composition after acute weight loss with a VLCD.

Variables	Total	*p* value
WL (kg)	(-) 6.3 ± 2.69 (2.0-15.0)	0.02*
WL (%)	(-) 4.78 ± 1.5 (1.53-10.7)	0.03*
Δ BMI (kg/m^2^)	(-) 2.4 ± 1.15	0.001*
Δ NC (cm)	(-) 1.1 ± 0.1	0.01*
Δ WC (cm)	(-) 2.0 ± 0.1	0.01*
Δ HC (cm)	(-) 2.1 ± 0.18	0.02*
Δ BF (kg)	(-) 4.9 ± 0.31	0.03*
Δ BF (%)	(-) 1.6 ± 0.19	0.01*
Δ FFM (kg)	(-) 0.6 ± 0.21	0.02*
Δ FFM (%)	(-) 1.6 ± 0.19	0.01*

Note: WW: weight loss; BMI= body mass index; NC= neck circumference; WC= waist circumference; HC= hip circumference; BF: body fat; FFM: fat-free mass; Δ= alteration. *p<0.05.

**Table 3:** Body composition alteration after rapid weight loss with VLCD according to gender, age, initial BMI and diabetes diagnosis.

	Men	Women	*p*	Age ≥60 years	Age <59 years	*p*	BMI≥50 kg/m^2^	BMI<49 kg/m^2^	*p*	DM	No DM	*p*
	n= 29	n= 91		n=9	n=111		n=54	n=66		n=35	n=85	
Weight (kg)	(-)6.7±0.34	(-)6.0±0.31	0.6	(-)7.4±0.98	(-)6.1±0.25	0.11	(-)6.5±0. 36	(-)6.0±0.33	0.11	(-)6.0±0.44	(-)6.3±0.30	0.34
Weight (%)	(-)4.6±0.27	(-)4.9±0.39	0.07	(-)6.0±3.71	(-)4.6±4.07	0.03*	(-)4.6±0.34	(-)5.0±0.59	0.34	(-)4.7±0.41	(-)4.7±0.20	0.69
BMI (kg/m^2^)	(-)2.3±0.12	(-)2.5±0.12	0.07	(-)2.8±0.41	(-)2.3±0.10	0.09	(-)2.5±0.15	(-)2.3±0.13	0.19	(-)2.3±0.18	(-)2.4±012	0.76
NC (cm)	(-)1.5±0.19	(-)1.0±0.12	0.19	(-)1.0±0.3	(-)1.2±0.10	0.72	(-)1.2±0.15	(-)1.0±0.13	0.37	(-)1.0±0.17	(-)1.2±0.12	0.59
WC (cm)	(-)1.5±0.43	(-)2.0±0.23	0.86	(-)3.0±0.83	(-)2.0±0.21	0.17	(-)1.8±0.31	(-)2.0±0.27	0.92	(-)2.0±0.39	(-)2.0±0.24	0.84
HC (cm)	(-)2.2±0.38	(-)2.0±0.2	0.64	(-)3.0±0.55	(-)2.0±0.19	0.33	(-)2.0±0.26	(-)2.7±0.25	0.42	(-)2.0±0.30	(-)3.0±0.22	0.17
BF (kg)	(-)5.7±0.48	(-)4.5±0.38	0.19	(-)6.8±1.31	(-)4.8±0.32	0.26	(-) 5.5±0.51	(-)4.5±0.28	0.52	(-)4.4±0.58	(-)4.9±0.37	0.98
BF (%)	(-)1.7±0.32	(-)1.5±0.22	0.48	(-)1.6±0.58	(-)1.2±0.20	0.56	(-)1.4±0.28	(-)1.7±0.24	0.12	(-)1.6±0.38	(-)1.6±0.21	0.31
Δ FFM (kg)	(-)0.2±0.38	(-)0.8±0.25	0.34	(-)1.1±0.73	(-)0.5±0.22	0.84	(-)1.0±0.35	(-)0.6±0.25	0.89	(-)0.8±0.34	(-)0.8±025	0.24
Δ FFM (%)	(-)1.7±0.32	(-)1.5±0.22	0.48	(-)1.5±0.58	(-)1.9±0.20	0.56	(-)1.4±0.28	(-)1.5±0.23	0.12	(-)1.6±0.31	(-)1.6±0.21	0.31

Note: DM: diabetes mellitus; BMI: body mass index; NC: neck circumference, WC: waist circumference; HC: hip circumference; BF: body fat; FFM: fat-free mass; TBW: total body water. *p<0.05.
